# The efficacy and safety of a new fixed-dose combination of amodiaquine and artesunate in young African children with acute uncomplicated *Plasmodium falciparum*

**DOI:** 10.1186/1475-2875-8-48

**Published:** 2009-03-16

**Authors:** Sodiomon B Sirima, Alfred B Tiono, Adama Gansané, Amidou Diarra, Amidou Ouédraogo, Amadou T Konaté, Jean René Kiechel, Caroline C Morgan, Piero L Olliaro, Walter RJ Taylor

**Affiliations:** 1 Centre National de Recherche et de Formation sur le Paludisme (CNRFP), BP 2208, Ouagadougou, Burkina Faso; 2 Groupe de Recherche Action en Santé, Ouagadougou, Burkina Faso; 3 Drugs for Neglected Diseases *initiative (DNDi)*, Geneva, Switzerland; 4 Cardinal Systems, Paris, France; 5 UNICEF/UNDP/WB/WHO Special Programme for Research & Training in Tropical Diseases (TDR), Geneva, Switzerland; 6 Service de Médecine Internationale et Humanitaire, Hopitaux Universitaries de Genève, Geneva, Switzerland

## Abstract

**Background:**

Artesunate (AS) plus amodiaquine (AQ) is one artemisinin-based combination (ACT) recommended by the WHO for treating *Plasmodium falciparum *malaria. Fixed-dose AS/AQ is new, but its safety and efficacy are hitherto untested.

**Methods:**

A randomized, open-label trial was conducted comparing the efficacy (non-inferiority design) and safety of fixed (F) dose AS (25 mg)/AQ (67.5 mg) to loose (L) AS (50 mg) + AQ (153 mg) in 750, *P*. *falciparum*-infected children from Burkina Faso aged 6 months to 5 years. Dosing was by age. Primary efficacy endpoint was Day (D) 28, PCR-corrected, parasitological cure rate. Recipients of rescue treatment were counted as failures and new infections as cured. Documented, common toxicity criteria (CTC) graded adverse events (AEs) defined safety.

**Results:**

Recruited and evaluable children numbered 750 (375/arm) and 682 (90.9%), respectively. There were 8 (AS/AQ) and 6 (AS+AQ) early treatment failures and one D7 failure (AS+AQ). Sixteen (AS/AQ) and 12 (AS+AQ) patients had recurrent parasitaemia (PCR new infections 10 and 6, respectively). Fourteen patients per arm required rescue treatment for vomiting/spitting out study drugs. Efficacy rates were 92.1% in both arms: AS/AQ = 315/342 (95% CI: 88.7–94.7) vs. AS+AQ = 313/340 (95% CI: 88.6–94.7). Non-inferiority was demonstrated at two-sided α = 0.05: Δ (AS+AQ – AS/AQ) = 0.0% (95% CI: -4.1% to 4.0%). D28, Kaplan Meier PCR-corrected cure rates (all randomized children) were similar: 93.7% (AS/AQ) vs. 93.2% (AS+AQ) Δ = -0.5 (95% CI -4.2 to 3.0%). By D2, both arms had rapid parasite (F & L, 97.8% aparasitaemic) and fever (97.2% [F], 96.0% [L] afebrile) clearances.

Both treatments were well tolerated. Drug-induced vomiting numbered 8/375 (2.1%) and 6/375 (1.6%) in the fixed and loose arms, respectively (*p *= 0.59). One patient developed asymptomatic, CTC grade 4 hepatitis (AST 1052, ALT 936). Technical difficulties precluded the assessment and risk of neutropaenia for all patients.

**Conclusion:**

Fixed dose AS/AQ was efficacious and well tolerated. These data support the use of this new fixed dose combination for treating *P. falciparum *malaria with continued safety monitoring.

**Trial registration:**

Current Controlled Trials ISRCTN07576538

## Background

Drug-resistant *Plasmodium falciparum *has rendered many anti-malarial drugs ineffective with a consequential increase in childhood morbidity and mortality, especially in Africa [1-3]. Many countries, most of them in Africa, are now using artemisinin-based combination therapy (ACT) following the demonstration of their superiority over standard monotherapies and the subsequent recommendation by the World Health Organization (WHO) [4,5].

The rationale for using ACT is based on the concept that the artemisinin will substantially and rapidly reduce even multidrug-resistant *P. falciparum *parasitaemia, leaving the residual parasitaemia to be killed by high concentrations of the partner drug. In this way, the probability of the development of *de novo *resistance is greatly reduced [6]. ACT also reduces gametocyte carriage and infectivity [5,7,8]. Artesunate and mefloquine has reduced *P*. *falciparum *transmission in the low transmission areas of the Thai-Burmese border [9] and artemether/lumefantrine (Coartem^®^) has also contributed to transmission reduction in KwaZulu Natal [10].

The WHO considers rapid and effective treatment with an ACT essential in its quest to roll back malaria [4]. Another element considered essential is treatment compliance. There is increasing acceptance that compliance is improved by the use of simplified, fixed-dose combinations presented in easy-to-use packaging [11,12]. Currently, the combination treatment artemether/lumefantrine is the only fixed dose combination registered to international standards that is widely available in malaria-endemic countries, but artesunate/mefloquine has been registered recently in Brazil and other combinations are under development, notably, dihydroartemisinin/piperaquine and artesunate/pyronaridine.

The efficacy of amodiaquine (AQ) combined with artesunate (AS) varies in Africa, achieving high (≥ 90%) cure rates in some countries [13-16] but lower rates in countries such as Kenya (80%), Rwanda (80%), and Tanzania (89%) [13,17,18]. In Burkina Faso, where the failure rate of chloroquine was 81% by Day (D) 28 in a previous trial [19], two small studies have evaluated loose AS combined with AQ (AS+AQ). The cure rates of AS+AQ were 100% in 33 children aged 1 to 15 and 82% in 61 children aged 6 to 10 years [20,21].

AS+AQ is generally well tolerated as treatment [13], but as malaria prophylaxis in travellers, AQ alone caused severe neutropaenia and severe hepatitis [22-24]. This calls for vigilance now that some countries are using AQ+AS widely, because repeat treatment is often required. To date, there are few data on the risk of AQ (alone or with AS) inducing severe neutropaenia (<1,000/microL) when treating *P. falciparum *malaria. In one trial, asymptomatic neutropaenia (<1,000/microL) on Day 28 occurred in 6% of 153 AQ alone or AQ+AS-treated, young African children who had normal D0 neutrophil counts [13]. In a meta-analysis of circa 5,000 patients treated with AQ, there were no AQ-related deaths (Olliaro, P unpublished data).

The present study was conducted as part of the clinical development of a new fixed-dose combination of amodiaquine and artesunate that aimed to make available a new, inexpensive, simple, and blister-packed dosing regimen based on age [25]. Several blister packs of loose AS and AQ are used currently in several countries in West Africa, including Burkina Faso. Given that the transition in drug policy from a loose to a fixed-dose combination would be an easier option for Ministries of Health, if the latter proved successful, it was decided to test the fixed-dose against the loose-dose combination of AS and AQ in children five and under, the most vulnerable group for malaria.

## Methods

### Study design

This randomized, controlled, open-label study compared the efficacy, using a non-inferiority design, and safety of a new, fixed-dose combination of oral artesunate and amodiaquine (AS/AQ), to the loose dose combination (AS+AQ), currently used in Burkina Faso. The study was carried out in two medical centres in the Koupela health district of Burkina Faso over two malaria seasons from October 2004 to February 2006.

The study was approved by the Burkina Faso ethics committee for health research and the ethics committee of the WHO and was carried out in accordance with the International Conference on Harmonization (ICH) Guidelines for Good Clinical Practice. Written informed consent was obtained from parents or guardians prior to patients commencing treatment. This trial is registered at .

### Study conduct

Patients of both sexes, aged between six months and five years, weighing ≥ 5 kg, with *P. falciparum *monoinfection of >1,000 parasites per μL and a measured fever (axillary temperature ≥ 37.5°C), were included in the study. Patients were excluded if they: (i) had features of severe and complicated malaria [26], (ii) had taken the study drugs or other drugs for malarial treatment within seven days prior to inclusion (or within three days if an artemisinin), or (iii) if they were receiving treatment of antibiotics with anti-malarial activity.

A randomization list was generated by a computer in blocks of 50. Individual treatment allocations were kept in sealed envelopes and opened after patients were admitted into the study. All treatments were administered by a study nurse. AS/AQ was administered according to a newly designed, age-based dosing regimen [25], and AS+AQ according to the manufacturer's instructions for the Arsucam^® ^blister pack (sanofi-aventis, Paris, France). For both regimens, the target doses for AS and AQ base are 4 mg per kg per day and 10 mg per kg per day, respectively, with newly defined, therapeutic ranges of 2 to 10 mg per kg per day and 7.5 to 15 mg per kg per day.

The fixed tablet contained 25 mg of AS and 67.5 mg of AQ. The dose was one (aged <12 months) or two (aged 12 to 60 months) tablets. The loose blister pack contained 50 mg tablets of AS (Arsumax^® ^, sanofi-aventis) and 153 mg tablets of AQ (Flavoquine^® ^, sanofi-aventis). The dose was half or one tablet of both drugs for the ages, as described above. Patients remained in the health centre for an hour following each treatment administration. Vomiting during this observation period resulted in re-administration of the same dose of study drugs.

Following the first visit (D0), patients were seen after 24 hours (D1), 48 hours (D2), 72 hours (D3), 7 days (D7), 14 days (D14), 21 days (D21) and 28 days (D28) for clinical (symptoms, temperature) and parasitological (Giemsa-stained thick films and PCR blots [except D1 for both]) assessments. Parasite density was determined by counting the number of asexual parasites per 200 leucocytes on a Giemsa-stained thick film and expressed as the number per μL, assuming a leucocyte count of 8,000 per μL. If up to 500 parasites had been counted before reaching 200 leucocytes, the counting process was stopped at the end of the last field. Gametocytes were counted and expressed as the number per 1,000 leucocytes (thick film). Routine haematology (Pentra 60^® ^) and biochemistry (Hospitex Screen Master Tecno^® ^) blood samples were taken and analysed on D0, D7, and D28.

Patients failing or not tolerating their treatment were withdrawn from the study and rescued with 25 mg per kg per day of oral or parental quinine base in three divided doses for seven days. There was a systematic investigation of all patients lost in follow-up.

### Primary endpoint

The primary efficacy endpoint was the PCR-corrected parasitological cure rate (also known as Adequate Clinical and Parasitological Response [ACPR]) based on a per protocol defined analysis population (see below). The criteria for treatment failure followed broadly those of the WHO [27]: (i) signs of severe malaria or danger signs at any time during follow up, (ii) parasitaemia at D2>D0, (iii) D3 parasitaemia ≥ 25% of that measured on D0, (iv) D7 parasitaemia, and (v) a recurrent parasitaemia after D7 that was a PCR-proven, recrudescent parasitaemia.

Patients with post-D7 recurrent parasitaemia were classed as either failure or new infections by analysing sequentially three parasite genes by PCR: first the merozoite surface protein 2 (*msp2*), then merozoite surface protein 1 (*msp1*), and then glutamine-rich protein (*glurp*), according to a previously published method [19]. A new infection was diagnosed if the allelic pattern for any one of the loci differed completely between the baseline and recurrent samples. All other allelic patterns were diagnosed as resistant infection.

### Secondary endpoints

These were: (i) the D28 crude (i.e. not corrected by PCR) parasitological cure rates, (ii) the proportions of patients without parasitaemia on D2 and D3, (iii) the proportions of patients with fever on D2 and D3, and (iv) the proportions of patients with gametocytes during follow-up.

### Safety

Safety was assessed by collecting clinical (symptoms, signs) and routine laboratory data during follow up. An adverse event was defined as a new symptom or sign that developed post treatment or the exacerbation of a symptom or sign present at baseline. A serious adverse event (SAE) was one resulting in hospitalization, significant disability or death. AE severity was determined using the Common Toxicity Criteria (US NIH, CTC v2 1999) and graded as mild, moderate, severe, or very severe.

### Sample size and statistical analysis

Sample size was calculated to demonstrate the non-inferiority in efficacy of AS/AQ compared to AS+AQ at a one-sided 5% α-level and a power of 90%. Assuming an efficacy of 95% on the loose combination and a maximum difference of 5% for the combination therapy, the calculated sample size was 326 patients per group. Estimating a 15% reduction in sample size (e.g. loss to follow-up), the sample size to be recruited was 374 patients per arm. Although the study was only designed to demonstrate non-inferiority at a one-sided 5% α-level, at the time of analysis, non-inferiority was also demonstrated (upper bound of the CI for the difference in cure rates <5%) using a more stringent two-sided 5% α-level. Therefore, this article reports the more stringent 95% CI.

Data were managed and analysed by an external clinical research organization, according to a predefined analysis plan. The per-protocol dataset excluded randomized patients who: (i) did not meet study entry criteria, (ii) took drugs with anti-malarial activity while on study, (iii) were lost to follow up or withdrew consent. In this analysis, patients withdrawn from the study for drug-induced AEs and those who persistently spat out the study drug were counted as treatment failures. PCR-determined new infections were counted as cured.

A supportive analysis was carried out to assess the primary end point on all randomized patients using the Kaplan-Meier product limit estimates of survival, an analysis recommended by the WHO. PCR-defined resistant or new infections and other study withdrawals (e.g. drug induced AEs, protocol deviations) were censored at the time of withdrawal. Patients lost to follow up were censored on the last day they were seen. Several other preplanned sensitivity analyses were carried out for robustness. Proportional and continuous data were analysed with Chi-squared (or Fisher's exact) and Wilcoxon tests, respectively.

## Results

**Figure 1:**
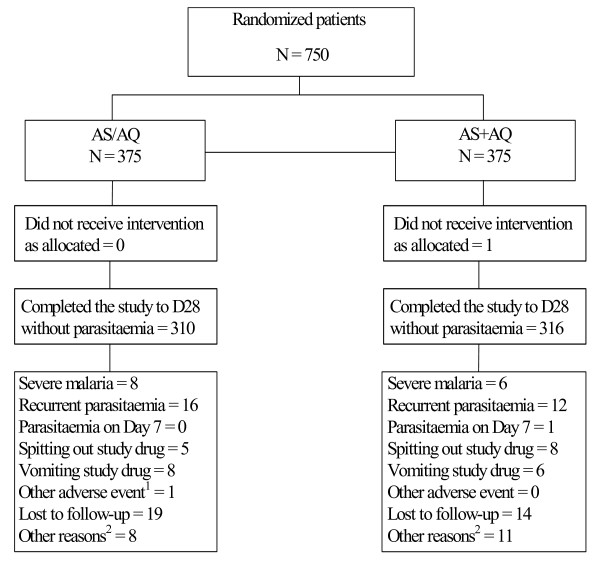
**Patient disposition**. 1. SAE: 8 month old girl hospitalized for gastroenteritis. 2. Other reasons were: parent's/guardian's decision; patients taking anti-malarial drugs during follow up.

A total of 750 patients were enrolled and randomized in the study, 375 per arm. Of these, 626 patients (83.5%) completed the study to D28. A total of 65 patients treated with AS/AQ and 59 patients treated with AS+AQ withdrew or were withdrawn from the study) (Figure 1).

### Baseline characteristics & doses administered

shows the baseline characteristics of all recruited children. Of the randomized patients, 345 (46.0%) were females and 405 (54.0%) males. The mean (SD) age of patients at baseline was 27.53 (14.72) months. There were no significant differences between treatment groups at baseline for all variables studied, including demography (age, sex, and ethnic group), clinical variables (weight, temperature, and palpable spleen and liver), and the measured parasitic variables and laboratory parameters, confirming adequate randomization.

The median doses of both drugs administered are shown in Table 2. Based on the newly defined therapeutic dosing ranges for AS and AQ, all patients except one (fixed arm) received a therapeutic dose of AS and 293 (78.5%, fixed group) and 217 (57.9%, loose) received AQ doses within the range (*p *< 0.0001). In the fixed group, 4 (1.1%) and 76 (20.4%) were dosed below and above the AQ range, respectively; the corresponding figures for the loose group were 0 and 158 (42.1%). Children under 12 months of age received lower doses of both AS and AQ compared to those aged 1 to 5 years (*p *< 0.0001).

**Table 1:** Baseline characteristics of patients receiving fixed or loose artesunate and amodiaquine

	**AS/AQ**	**AS+AQ**	**p-value**
Male: n (%)*	193 (51.5)	212 (56.5)	0.16
Age (months): Mean (SD)	27.5 (14.2)	27.5 (15.2)	0.78
< 12 m	n = 54	n = 59	
1–5 y	n = 321	n = 316	
Weight (kg): Mean (SD)	10.5 (3.0) n = 373	10.4 (3.1)	0.66
Temp (°C): Mean (SD)	38.4 (0.8)	38.4 (0.9)	0.52
Splenomegaly: n (%)	37 (9.9)	38 (10.1)	0.90
Parasite count (/μL): Median (range)	14.5 (1 – 516.6)	14.2 (1 – 410)	0.78
Haemoglobin (g/dL): Mean (SD)	8.3 (1.7) n = 374	8.2 (1.7) n = 371	0.50
Total WCC (10^9/L): Mean (SD)	11.5 (5.1) n = 337	11.6 (4.8) n = 337	0.42
Platelet count (10^9/L): Mean (SD)	174.4 (112.6) n = 337	183.9 (113.4) n = 338	0.20
AST (IU/L): Mean (SD)	71.0 (71.1) n = 303	70.0 (76.3) n = 298	0.53
ALT (IU/L): Mean (SD)	31.8 (44.9) n = 299	32.0 (53.1) n = 293	0.87
Total bilirubin (μmol/L): Mean (SD)	26.0 (27.8) n = 321	25.7 (33.9) n = 311	0.77

* n = 375 unless stated

**Table 2:** Median (range) doses of artesunate and amodiaquine, expressed as mg/kg, administered on Day 0

	**AS/AQ (fixed) N = 373**		**AS+AQ (loose) N = 375**	
	
Age*	**AS** **median (range)**	**AQ** **median (range)**	**AS** **median (range)**	**AQ** **median (range)**
**0–11 months**	3.57 (2.50 – 5.56) N = 54	9.64 (6.75 – 15.00) N = 54	3.57 (2.50 – 6.85) N = 59	10.86 (7.60 – 20.96) N = 59
				
**12+ months**	4.67 (1.79 – 10.00) N = 319†	12.62 (4.82 – 27.00) N = 319†	4.72 (2.50 – 10.00) N = 316	14.44 (7.65 – 30.60) N = 316

* Median doses for AS and AQ were significantly (p < 0.0001) higher comparing children <12 vs. ≥ 12 months.

† The weights of 2 patients (19 months old and 42 months old) assigned to the AS/AQ treatment group were not available. Both patients received 2 tablets at 25/67.5 mg each on D0

### Efficacy analyses

Fourteen patients (8 = fixed and 6 = loose) had early treatment failure due to the development of severe malaria or danger signs between D0 and D3. One patient in the AS+AQ group who was parasite-free on D2 and D3 became parasitaemic on D7. Twenty-eight patients had recurrent parasitaemia after D7: 16 from the AS/AQ group and 12 from the AS+AQ group. Of these, 10 (AS/AQ) and 6 (AS+AQ) patients were classed by PCR as new infections and 6 from each arm were classed as recrudescent infections.

### Primary analysis

**Figure 2:**
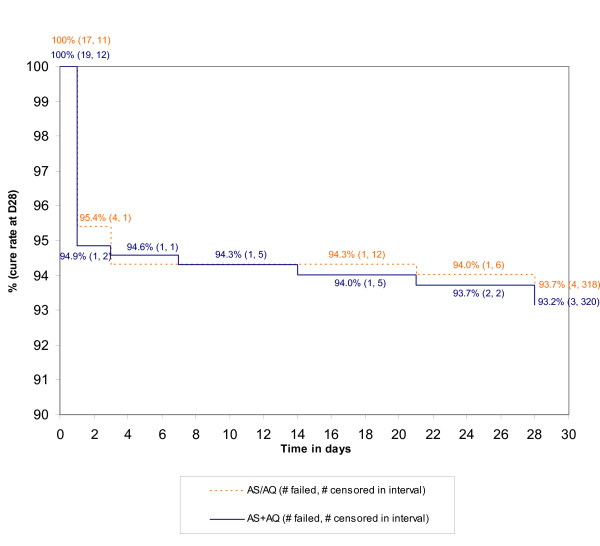
**Kaplan Meier survival efficacy analysis of all randomized patients**. Rates are expressed as 1 minus the rate of parasitological failure.

The PCR-corrected parasitological cure rates at D28 were 92.1% in both treatment groups, AS/AQ = 315/342 (95% CI: 88.7–94.7) vs. AS+AQ = 313/340 (95% CI: 88.6–94.7), Δ (AS+AQ – AS/AQ) = 0.0% (95% CI: -4.1% to 4.0%). Results for all analysis datasets studied are presented In Table 3. As shown in Figure 2, the Kaplan Meier cure rates at D28 were 93.7% (AS/AQ) and 93.2% (AS+AQ) with 95% CI (AS+AQ – AS/AQ) from -4.2% to 3.0% (Wald method). All pre-planned sensitivity analyses of the primary endpoint confirmed non-inferiority. The crude parasitological cure rates at D28 were 89.2% (AS/AQ) vs. 90.3% (AS+AQ) with 95% CI (AS+AQ – AS/AQ) from -3.4% to 5.7%.

**Table 3:** PCR-corrected cure rates on all predefined analysis datasets

**Data set**	**AS/AQ**	**AS+AQ**	**Difference (95% CI)** **AS+AQ – AS/AQ**
**PP dataset**	92.1% (315/342)	92.1% (313/340)	0.0% (-4.0%, 4.1%)
**Modified PP dataset**^1^	95.7% (315/329)	96.0% (313/326)	0.3% (-2.8%, 3.3%)
**Full dataset (ITT)**^2^	85.3% (320/375)	85.9% (322/375)	0.5% (-4.5%, 5.6%)

1. The modified PP dataset excluded patients vomiting/spitting out study drug in addition to patients already excluded from the PP dataset

2. Patient withdrawals due to vomiting/spitting out study drug, refusal to take study drug, loss to follow up, patients taking anti-malarial drugs during follow up, etc., were counted as failures for analysis purposes

The median doses of AS and AQ received in cured patients (N = 625) were 4.55 mg per kg and 12.75 mg per kg, respectively. These doses were significantly higher than the 28 patients with recurrent parasitaemia: 3.85 mg per kg (p = 0.05) and 11.77 mg per kg (p = 0.04). However, the differences were not significant for: (i) PCR resistant infections (N = 12): 3.85 mg/kg (p = 0.18) and 11.77 mg/kg (p = 0.21), and (ii) PCR new infections (N = 16): 3.98 mg per kg (p = 0.15) and 11.66 mg per kg (p = 0.10).

**Figure 3:**
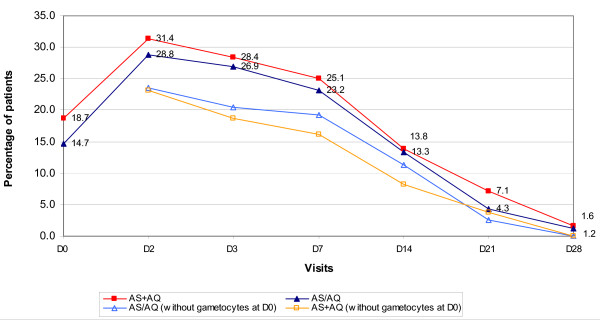
**Proportion of patients with gametocytes at baseline and during follow up**.

No significant differences were observed between the two treatment groups in terms of the proportions of patients: (i) aparasitaemic on D2 (F: 339/347 [97.7%] *vs. *L: 337/344 (98.0%), p = 0.81] or (ii) afebrile on D2 [F: 337/347 (97.1%) *vs*. L: 331/344)96.2%), p = 0.51], and (ii) with gametocytes during follow up (Figure 3).

### Clinical adverse events

Both drug regimens were well-tolerated. Through direct questioning, 76.3% (286 of 375) of patients in the AS/AQ group reported at least one AE over 28 days, which was similar (*p *= 0.61) to the percentage of patients reporting AEs in the AS+AQ group, 74.7% (280 of 375). None of these were probably or definitely related to the study treatment. The number of patients with at least one possibly related solicited AE was similar among the two groups (AS/AQ group: 22 of 375 [5.9%] vs. AS+AQ group: 21 of 375 [5.6%]). The most frequently solicited AEs were coughing, rhinitis, anorexia, diarrhoea and abdominal pain. In total, 14 of 750 (1.9%) patients were withdrawn from the study for drug-induced vomiting, 8 (2.1%) and 6 (1.6%) in the fixed and loose arms, respectively (*p *= 0.59). Of the 14 withdrawn, one received an AQ dose below the therapeutic range, seven received AQ doses within the therapeutic range, and six received AQ doses above the therapeutic range; doses in the six were (i) fixed: 16.9, 21.1 mg per kg per day, and (ii) loose: 15.3, 17, 19.1, and 23.5 mg per kg per day.

Nine patients experienced a total of 12 SAEs, including two (0.27%) deaths. All SAEs occurred within the first three days of treatment except for one patient, who had gastroenteritis requiring rehydration in the hospital on D11. Four of these patients (1.1%) were in the AS/AQ group and five (1.3%) in the AS+AQ group (p = 0.74). In the AS/AQ group these were: (i) one death on D1 owing to severe malaria (D0 parasitaemia 11,224/μL), (ii) two patients with convulsions (severe malaria) and (iii) one child with gastroenteritis. AS+AQ SAEs were: (i) one death on D1 owing to severe malaria (D0 parasitaemia 1651/μL), (ii) one patient with convulsions and anaemia secondary to severe malaria, (iii) one patient with severe prostration (severe malaria), and (iv) two patients with acute respiratory distress, consistent with malaria-induced metabolic acidosis or pneumonia. All SAEs were considered unrelated to the study drugs.

### Laboratory parameters

Because the mean values and mean changes from baseline were very similar in both treatment groups, laboratory data from both arms have been combined. By D28, the mean haematocrit increased from 26.6 to 31.2% (*p *< 0.0001). The mean platelet counts rose from 179.2 to 337.6 × 10^9^/L by D28 (p < 0.0001).

During the first malaria season (2004), the Pentra 60^® ^machine at the Centre National de Recherche et de Formation sur le Paludisme (CNRFP) broke down, resulting in the full blood count (FBC) being determined at a private laboratory, where the differential white cell counts (WCC) were determined manually in a total of 350 patients. The results are reported here for completeness, but are deemed to be inaccurate because of the clear difference in the rates of neutropaenia (defined as a neutrophil count of <1,000 per μL) and leucopaenia (WCC <6,000 per μL for children <12 months, <5,000 per μL for children between one and five years of age) between the two laboratories. The total number of children (2004 and 2005 combined) with D0 and D28 neutropaenia and D0 and D28 leukopaenia were: (i) 32 of 657 (4.9%) and 87 of 569 (15.3%), (ii) 22 of 674 (3.3%) and 34 of 569 (6.0%), respectively. Of these totals, 28 of 32 (87.5%), 85 of 87 (97.7%), 15 of 22 (68.2%) and 33 of 34 (97.1%) were reported in 2004 by the private laboratory. All children with reported neutropaenia (2004 or 2005) were well; two were mildly febrile and only required symptomatic treatment.

Compared to D0, the mean D28 AST (70 vs. 60 IU per L, *p *= 0.02) and D28 total bilirubin (25.85 vs. 13.1 μmol per L, *p *< 0.0001) values fell significantly. Mean serum ALT (32 vs. 29 IU/L) and creatinine (37 vs. 38 μmol per L) values changed little over time. Six of 529 (1.1%) patients with D28 ALT results had CTC grade 2 raised (>2.5 to 5 × ULN) ALT concentrations (range 104 to 196 IU per L) and one 2-year-old child (0.19%) in the AS+AQ group had asymptomatic, CTC grade 4 hepatitis [AST 1052, ALT 936 IU per L]). All seven children had normal ALT values at baseline, were clinically well and aparasitaemic with normal D28 total bilirubin values, but all had raised D28 AST (range 73 to 1052 IU per L).

## Discussion

This study has shown that amodiaquine and artesunate taken as either a fixed- or a loose-dose combination were highly effective for the treatment of acute, paediatric, uncomplicated *P. falciparum *malaria. Efficacy rates were ~93% for both combinations and rose to 96% when children who vomited/spat out their drugs were excluded, exceeding the WHO-recommended 90% efficacy rate for anti-malarial treatments [11]. Both combinations were well tolerated with only 2% of children discontinuing because of drug-induced vomiting.

The fixed dose regimen was compared with the loose combination for three principal reasons. There was already experience with the loose formulation in West Africa. At the time of the study, chloroquine was the recommended first-line treatment in Burkina Faso, but this failing drug could not be used as a comparator. Another option could have included the registered artemether/lumefantrine. However, this combination did not then have a formulation that was either suitable or registered for children less than 10 kg and would have necessitated their exclusion from this study.

This new fixed-dose combination used a new, simplified, computer-modelled, age-based regimen that was blister packed [25]. Making anti-malarial drug regimens user friendly may have a positive effect on treatment adherence [12], a crucial element in reducing the development and spread of drug resistance caused by sub-therapeutic drug concentrations [28]. Interestingly, the fixed dose was better for overall dosing accuracy than the loose regimen, which is used currently in several African countries. In this study, the fixed regimen resulted in 1/5 of children receiving an AQ dose in excess of the upper therapeutic range but no significant effect on tolerability was seen. The proportions of under-dosed patients were small (0.3% AS, 1.1% AQ), but this may have a role in the development of drug resistance at a population level and must be investigated as part of any long-term studies.

Comparing the Burkina Faso children who participated in this trial with the 88,000 African children up to five years of age in the modelled, fixed-dose database [21], the median doses received daily of AS (3.6 vs. 3.6 mg/kg [<12 m], 4.7 vs. 4.1 [children 1–5 years old], respectively) and AQ ([9.7 vs. 9.8 mg per kg], 12.2 vs. 11.2 mg per kg, respectively) and those who received excess AQ doses (20.4 vs. 19%) were close, but the proportions of AQ recipients underdosed was less in the Burkina Faso children (1.1% vs. 13.9%). The difference in the latter is probably due to a lower rate of heavier children in the study. This is the first clinical trial to assess the new, age-based, fixed-dose combination; it is encouraging that the dosing data in this study are similar to those predicted by the model. The median doses of both drugs received in cured patients were higher compared to patients with recurrent parasitaemia, but significance was lost when stratified by recrudescent and new infections. These data raise interesting questions regarding dose and outcome. More detailed analyses are planned.

The efficacy of AS and AQ depends on the level of background resistance to amodiaquine, which is also partially cross-resistant to chloroquine *in vitro *[29,30]. All degrees of chloroquine resistance are present in Burkina Faso and clinical chloroquine resistance in Burkina Faso children was 81% in a recent trial [19]. There are no current *in vitro *resistance data for AQ from Burkina Faso and a small study of 22 AQ-treated children (aged 1 to 15) found no clinical resistance. Based on the results of this study, clinical resistance to AQ in children under five is probably low.

The cure rate in this study compares well with AS+AQ in Angola, Sudan, the Democratic Republic of Congo, southern Senegal, Colombia, and Uganda, where cure rates exceeding 90% have been reported [16,31-35]. Nevertheless, lower rates have also been reported from Eastern Africa where alternatives to AS and AQ are being used [13,17,18]. This calls for continuing resistance monitoring of AS and AQ in countries that currently deploy or will deploy AS/AQ. In common with others, ASAQ was assessed over 28 days, but the cure rate may have been overestimated compared with 42 days of follow up [36].

Consistent with other trials, symptom and fever resolution were rapid. By D2, less than 3% of children were still parasitaemic and less than 4% were still febrile. Similarly, within one week, all but one child was asymptomatic. Gametocyte carriage at baseline was higher than the ~10% observed in an earlier trial in the same areas [19], reached a peak of ~30% and declined slowly over time. This propensity to produce gametocytes may have been related to anaemia and has appeared to counter the usually good antigametocytogenic effect of the artesunate [5,13]. Studies have also shown a reduction in mosquito infectivity consequent to reduced gametocyte carriage following treatment with artemether/lumefantrine and AS plus chloroquine or sulphadoxine/pyrimethamine [7,8]. The effect on transmission of the long-term use of ACT in hyper/holoendemic Africa is unknown and should be investigated.

AS+AQ was well-tolerated; only nine children suffered serious adverse events, which were all due to either the development of severe malaria or intercurrent illnesses. Two of the nine children died because of severe malaria, giving a case fatality rate of 0.27%. All children were assessed carefully on admission and the two children who died had low, baseline parasite counts. In such small children, a number may have been developing clinically silent features of severe malaria when first seen and their parasite counts may have been increasing despite the use of the rapidly acting artesunate. The parasitaemia threshold of 1,000/μL used is lower than the 2,000/μL recommended by the WHO for areas of intense malaria transmission and the parasite counts in children were consistent with many similar studies conducted in Africa. Nevertheless, as the use of ACT increases, trying to identify patients at risk of developing severe malaria may be a new avenue of research. Just over 2% of patients in this study suffered from AEs considered to be study drug related; most were due to drug-induced vomiting. A number of children persistently spat out their tablets, making drug administration challenging. Whilst this is not an AE *per se*, strategies for reducing drug refusal should be explored. No child in the fixed combination group developed itching and, consistent with other studies (<1 to 2%), only two children (0.3%) reported itching in the loose combination group [13,16]. This provides a contrast to some reports of the structurally related chloroquine, which has caused itching and/or an unpleasant prickly sensation in up to 20–67% of patients [37-39].

In this study, there was a downward trend in the mean AST and the total bilirubin, consistent with disease resolution, but mean ALT levels were stable over time, suggesting a small disease effect on this enzyme in these children. Using the D28 ALT values as a marker of possible AQ hepatocellular toxicity by study end, about 1% had moderate liver impairment and one child (0.19%) had severe biochemical hepatitis with liver enzymes of circa 1,000 IU per L. All of these children were well and none were jaundiced. These liver enzyme changes are also consistent with an intercurrent, viral-induced hepatitis, but serology was not measured. These findings have implications for the deployment of amodiaquine because repeat dose AQ as prophylaxis has caused severe hepatitis, sometimes in conjunction with neutropaenia [22]. AQ will certainly be used several times per year when deployed, especially in areas of intense malaria transmission. Ergo, amodiaquine safety will need to be assessed in well-designed studies of repeat use AQ.

The current study reported a total of 87 patients (15.3%) with neutrophil counts <1,000 per μL at D28 compared to 32 patients (4.9%) at D0. However, there was a significant difference between the differential white cell counts measured in 2004 and 2005. In 2004, the differential white cell count was done manually but after 2004, the count was done using a new Pentra 60^® ^machine. An audit of the external laboratory and a quality control of the differential white cell counts for a sample of the slides were carried out. It was concluded that the manual counts were not reliable and that the haematology data should be presented separately for 2004 and 2005. Consequently, the frequency of neutropaenia in children recruited in 2004 cannot be determined accurately. Taking the automated derived data from CNRFP, the rate of D28 neutropaenia (<1,000 per μL) was low (< 0.8%, n = 293). Nevertheless, continued monitoring for neutropaenia and abnormal liver function in studies involving amodiaquine should be done to document their risks of occurrence and their clinical features and significance.

## Conclusion

This new fixed-dose combination, dosed by age, was efficacious and well-tolerated. It is a good option for treating acute uncomplicated *P*. *falciparum *malaria and should be assessed in other malaria-endemic countries.

## Competing interests

The authors declare that they have no conflict of interest. This clinical study was funded by the Research Directorate General of the European Commission (Contract N°ICA4-CT-2002-10046) and the Drugs for Neglected Diseases *Initiative *(DND*i*). JRK was the DND*i *project manager and is a co-author on this paper, but had no direct part in the design or implementation of the trial. PO is a current staff member of the WHO and is required to declare that the opinions in this paper are not to be interpreted as those of the WHO. WRJT was a WHO staff member at the time of study conduct, but is not compromised by this past association.

## Authors' contributions

BSS, WRJT, and PO designed the study and developed the protocol. BSS, ABT, AG, AO, and AKT executed the study. WRJT and JRK oversaw and coordinated the study. CCM analysed the data. BSS, WRJT, JRK, CCM, and PO interpreted the data. BSS, WRJT, and CCM wrote the first draft of the paper. All co-authors have seen and approved the manuscript.
